# 

**Published:** 1910-11-15

**Authors:** 


### ANNOUNCEMENTS.

The New Jersey State Board of Registration and Examination in Dentistry will hold its annual meeting in the Assembly Chamber of the State House, Trenton, N. J., December 5th, 6th and 7th, 1910.

All applications must be in the hands of the Secretary ten days prior to the meeting.

For information apply to Charles A. Meeker, D.D.S., Secretary, 29 Fulton St., Newark, N. J.

Iowa Dental Examiners. This Board will meet on December 5th, in Des Moines, Iowa. For information, write to Dr. E. D. Brown, La Mars, Iowa.

Utah Dental Examiners. This Board will meet December 6th and 7th, in Salt Lake City. Address for particulars, Dr. A. C. Wherry, Salt Lake City, Utah.

